# Fate of methane in canals draining tropical peatlands

**DOI:** 10.1038/s41467-024-54063-x

**Published:** 2024-11-11

**Authors:** Clarice R. Perryman, Jennifer C. Bowen, Julie Shahan, Desi Silviani P.A.B, Erin Dayanti, Yulita Andriyani, Adibtya Asyhari, Adi Gangga, Nisa Novita, Gusti Z. Anshari, Alison M. Hoyt

**Affiliations:** 1 Department of Earth System Science, Stanford University, Stanford, CA USA; 2 Department of Soil Science, Tanjungpura University, Pontianak, Indonesia; 3 Magister of Environmental Science, Tanjungpura University, Pontianak, Indonesia; 4 Yayasan Konservasi Alam Nusantara, Jakarta, Indonesia; 5 The Nature Conservancy, Jakarta, Indonesia

**Keywords:** Carbon cycle, Geochemistry, Limnology, Carbon cycle

## Abstract

Tropical wetlands and freshwaters are major contributors to the growing atmospheric methane (CH_4_) burden. Extensive peatland drainage has lowered CH_4_ emissions from peat soils in Southeast Asia, but the canals draining these peatlands may be hotspots of CH_4_ emissions. Alternatively, CH_4_ oxidation (consumption) by methanotrophic microorganisms may attenuate emissions. Here, we used laboratory experiments and a synoptic survey of the isotopic composition of CH_4_ in 34 canals across West Kalimantan, Indonesia to quantify the proportion of CH_4_ that is consumed and therefore not emitted to the atmosphere. We find that CH_4_ oxidation mitigates 76.4 ± 12.0% of potential canal emissions, reducing emissions by ~70 mg CH_4_ m^−2^ d^−1^. Methane consumption also significantly impacts the stable isotopic fingerprint of canal CH_4_ emissions. As canals drain over 65% of peatlands in Southeast Asia, our results suggest that CH_4_ oxidation significantly influences landscape-scale CH_4_ emissions from these ecosystems.

## Abstract (web summary)

Canals draining vast areas of peatland in Southeast Asia may be hotspots for methane emissions. Perryman et al. surveyed dozens of canals in Indonesia and found that methane-eating microbes reduce emissions from these waterways by over 50%.

## Introduction

Wetlands and freshwaters contribute ~30–55% of global CH_4_ emissions^1^, with significant emissions from tropical ecosystems^2–4^. Rising CH_4_ emissions from tropical wetlands due to temperature and rainfall anomalies have contributed substantially to the growing atmospheric CH_4_ burden^5–8^. In addition to climate change, ongoing disturbances to tropical wetlands like deforestation, drainage, fertilizer application, and slash and burn agriculture, as well as rewetting and restoration efforts, stand to impact their contribution to the global CH_4_ budget^9–14^. However, the impact of tropical wetland disturbance on CH_4_ cycling is not well understood.

In Southeast Asia, wetland disturbance has heavily impacted peatlands, destabilizing the large pool of soil carbon stored in the peat soils of this region^15,16^. Peatlands in Southeast Asia have undergone extensive drainage for oil palm plantations, timber, and other agriculture through the construction of canals^17,18^ that lower the water table and therefore lower CH_4_ emissions from peat soils^19–21^. Instead, the CH_4_ produced in peat soils is transported into canals via lateral flow^22,23^, increasing the relative importance of drainage canals as a source of CH_4_ emissions^24,25^. Canals can represent over 50% of peatland CH_4_ emissions in Southeast Asia^26^, but estimates of the magnitude of drainage canal CH_4_ emissions vary by several orders of magnitude^27,28^. Given that drainage increases the importance of aquatic carbon fluxes from tropical peatlands^29^, and the large uncertainty around canal CH_4_ emissions, greater understanding of the key controls and mechanisms driving canal CH_4_ emissions is needed to constrain their role in tropical peatland CH_4_ budgets.

One process that strongly influences freshwater CH_4_ emissions is microbial oxidation of CH_4_ to carbon dioxide. In other tropical freshwaters (e.g., rivers, lakes) CH_4_ oxidation attenuates CH_4_ emissions by 40 to nearly 100%^23,30–32^. The fraction of CH_4_ transported into canals from drained peatlands that is oxidized instead of emitted is highly uncertain, as are the factors that mediate CH_4_ oxidation in drainage canals. For example, both aerobic and anaerobic methanotrophic microbiota are found in tropical freshwaters^32–35^. As variation in canal water depth and discharge can impact dissolved oxygen in canals^36,37^, examining the relationship between CH_4_ oxidation and dissolved oxygen could inform how CH_4_ oxidation in canal waters may vary over space and time. Constraining the importance of CH_4_ oxidation in canals draining tropical peatlands is a key step to improving our understanding of the processes controlling CH_4_ emissions from these ecosystems, especially in the densely drained peatlands of Southeast Asia where canals can have a disproportionate impact on landscape-scale CH_4_ emissions.

**Fig. 1:**
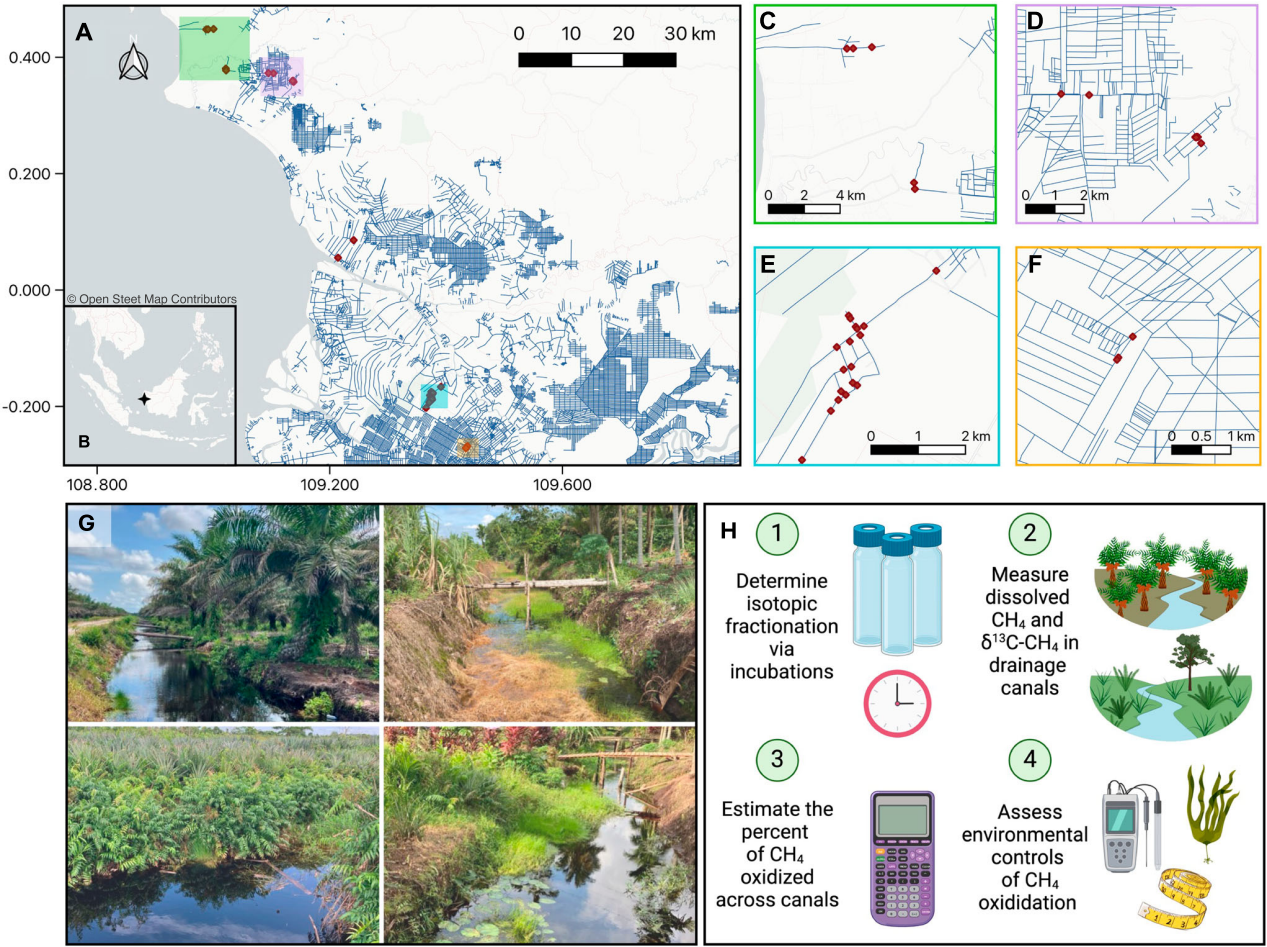
Drainage canal waters were collected in West Kalimantan, Indonesia to measure methane oxidation. **A** Study area with drainage canals shown as dark blue lines. **B** shows location of study within insular Southeast Asia. Canal sample locations marked in red points. **C**–**F** Zoomed in view of green, purple, teal, and orange boxes in panel **A**, respectively, showing sample locations. The base map layers in Panels **A**–**F** are available from OpenStreetMap (openstreetmap.org/copyright), available under the Open Database License. **G** Examples of canals in varying land use context and canals with and without aquatic vegetation. **H** Overview of study methods to estimate CH_4_ oxidation in drainage canals. Created in BioRender. Perryman, C. (2024) BioRender.com/c12r203.

Here, we address how much CH_4_ transported from drained tropical peatlands into canals is oxidized instead of emitted to the atmosphere. We quantified the percent of CH_4_ oxidized in 34 canal reaches that drain peat soils under varying land uses across West Kalimantan, Indonesia (Fig. 1, Supplementary Data 1) through shifts in the δ^13^C composition of CH_4_ during incubation experiments of canal waters and from field observations of in situ canal CH_4_ concentration and δ^13^C-CH_4_ (Fig. S1). We find that 47.3-91.3% of CH_4_ transported into canals from drained peatlands is oxidized instead of emitted. The fraction of CH_4_ that is oxidized is influenced by factors including dissolved oxygen, vegetation, and canal water depth. Overall, our results suggest that CH_4_ oxidation substantially attenuates CH_4_ emissions from canals, and as a result, may be a significant control of landscape-level CH_4_ emissions from drained peatlands in Southeast Asia.

## Results and Discussion

### CH_4_ consumption and isotopic fractionation observed during incubations

**Fig. 2:**
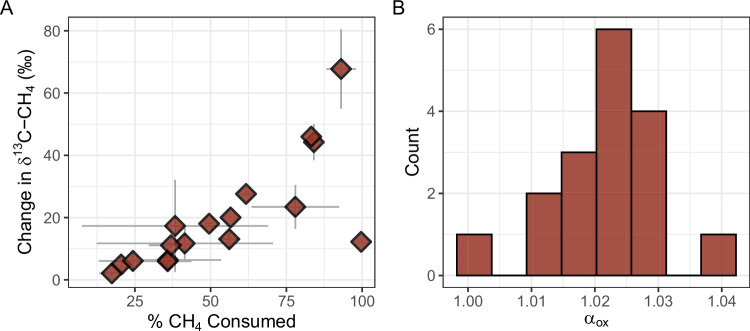
Methane consumption and resulting stable isotope fractionation in incubated canal waters. **A** Across incubated waters, δ^13^C-CH_4_ increased as the percent of initial CH_4_ consumed increased. Each data point shows the mean change over ~50 hours of incubation ± standard error of replicates (Table S1). **B** Histogram of α_ox_ values calculated from incubation data. Source data are provided as a Source Data file.

To confirm CH_4_ oxidation occurs in drainage canals, we incubated water from 13 canals at in situ dissolved CH_4_ and oxygen concentrations and measured the change in dissolved CH_4_ and δ^13^C-CH_4_ over time. On average, 53.8 ± 25.6% of the initial CH_4_ was consumed over the incubation period (17.6–99.7%) and δ^13^C-CH_4_ increased by 19.8 ± 17.7‰ (2.1–67.8‰, Fig. 2A, Table S1). The increase in δ^13^C-CH_4_ observed across incubated waters confirmed the loss of CH_4_ was from microbial oxidation, as CH_4_ oxidation leaves residual CH_4_ enriched in ^13^C^38,39^. Methane oxidation rates were variable across incubated waters (0.03–5.6 μmol CH_4_ L^−1^ d^−1^, Table S1) and were strongly influenced by initial CH_4_ concentration (Fig. S2). Neither CH_4_ production nor δ^13^C-CH_4_ depletion was observed in the canal waters during the incubation period.

From these data we calculated the first empirically derived isotopic fractionation factors for CH_4_ oxidation^40^ (α_ox_) in peat-draining freshwaters. Ecosystem-specific values for α_ox_ are critical to estimating the percent of CH_4_ that is oxidized rather than emitted from the natural environment^41,42^. Mean α_ox_ was 1.022 ± 0.009 across the incubated canal waters (range: 1.002–1.039; Fig. 2B). The range of α_ox_ encompasses past observations from northern and temperate freshwaters incubated under in situ dissolved CH_4_ and oxygen concentrations and temperature^42,43^, as well as results from incubations of soil from subtropical rice paddies^44^ (α_ox_ of 1.025–1.033) that are often used in estimates of CH_4_ oxidation in tropical freshwaters^30,32^. While CH_4_ oxidation rates varied with initial CH_4_ concentration, we did not observe a correlation between α_ox_ and initial CH_4_ concentration, nor α_ox_ and CH_4_ oxidation rate (Fig. S2). Recent work in temperate lakes identified temperature, pH, and dissolved O_2_ as potential controls on α_ox_^43^. Of these factors, α_ox_ was only weakly positively correlated with the initial dissolved O_2_ present in each of the incubated waters (*p* = 0.07). α_ox_ did not vary between surface and bottom waters of the subset of canals sampled at two depths for incubation experiments (1.024 ± 0.006 vs. 1.023 ± 0.012, *n* = 4 canals). As we did not find significant environmental correlates of α_ox_, we used the mean value to estimate in situ CH_4_ oxidation as discussed below.

### Oxidation mitigates the majority of drainage canal CH_4_ emissions

We find that the majority of CH_4_ transported into canals from drained tropical peatlands is oxidized instead of emitted to the atmosphere. Using the laboratory-derived α_ox_ values, measurements of in situ canal water δ^13^C-CH_4_ from 34 canal reaches (Supplementary Data 1), and measurements of source porewater δ^13^C-CH_4_ (Supplementary Data 2), we estimated that CH_4_ oxidation consumes 76.4 ± 12.0% of CH_4_ transported into canals (range: 47.3–91.3%). Considering the standard deviation of α_ox_ shifts the mean percent oxidized by ~10%, ranging from 65.5 ± 12.5% to 89.3 ± 8.9% (Figure. S3). Similarly, considering the standard deviation of the porewater source δ^13^C-CH_4_ measurements, the mean percent oxidized could range from 68.2 ± 16.1% to 82.4 ± 8.9% (Fig. S3). Our estimate of the fraction of CH_4_ transported into canals that is oxidized instead of emitted is consistent with Somers et al. (2023), who estimated that 70% of CH_4_ was oxidized in a canal draining a tropical peatland in Brunei using a reactive transport model. These results are also consistent with past work indicating that oxidation consumes ~80% of CH_4_ in blackwater rivers in the Amazon^32^ that have low pH and high concentrations of aromatic-rich, humic-like dissolved organic carbon like the canals in our study region^29,45^. Compared to previous work in lotic systems that use a similar approach as employed in our study, we find that oxidation mitigates a higher proportion of potential CH_4_ emissions in drainage canals than in headwater streams in temperate forests^46^ (55.6 ± 2.8%) or boreal peatlands^47^ (~60%).

**Fig. 3:**
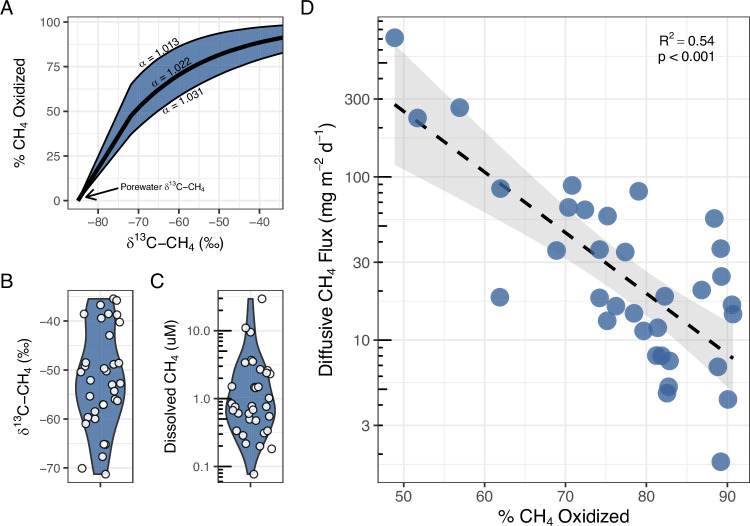
Survey of drainage canal CH_4_ concentrations and δ^13^C-CH_4_ reveal the impact of CH_4_ oxidation on canal CH_4_ emissions. **A** Curve showing the relationship between canal water δ^13^C-CH_4_ and estimated percent CH_4_ oxidized across the mean (black line) and ± 1 standard deviation (shaded region) of the laboratory derived α_ox_ value. **B**, **C** Surface water δ^13^C-CH_4_ and dissolved CH_4_ concentration across the studied canals (*n* = 34). **D** Estimates of the percent of CH_4_ oxidized versus estimated diffusive CH_4_ flux across the studied canals. For panels **B**–**D** each dot represents a canal. The shaded region of panel **D** represents the 95% confidence interval associated with the linear relationship. Dissolved CH_4_ concentration and estimated diffusive CH_4_ flux are shown on a log_10_ scale in panels **C** and **D**. Source data are provided as a Source Data file.

Canal water dissolved CH_4_ concentration and δ^13^C-CH_4_ across our study region supports our finding that CH_4_ oxidation limits CH_4_ release from canals. Dissolved CH_4_ concentration and δ^13^C-CH_4_ in canal waters ranged from 0.05 to 31.6 μM and −71.9 to −34.1‰, respectively (Fig. 3B), and dissolved CH_4_ decreased with increasing δ^13^C-CH_4_ (R^2^ = 0.43, *p* < 0.001, Fig. S4). Previous observations in tropical river networks^32^ also observed a negative relationship between the concentration of CH_4_ in river waters and δ^13^C-CH_4_. In these rivers δ^13^C-CH_4_ also had a positive relationship with gene markers for methanotrophic bacteria, indicating that variation in CH_4_ concentration and δ^13^C-CH_4_ is influenced by CH_4_ oxidation. The consistent relationship between CH_4_ concentration and δ^13^C-CH_4_ observed across the drainage canals in our study and these tropical rivers supports the idea that differences in dissolved CH_4_ concentrations between canal reaches are influenced by CH_4_ oxidation.

It is unlikely that CH_4_ concentration in canal waters is dictated only by the amount of CH_4_ originally transported into canals from the surrounding landscape, including CH_4_ produced in peat soils and canal sediments. Methane produced in ombrotrophic tropical peat soils is highly depleted in ^13^C^23,48^. Unlike in lakes where δ^13^C-CH_4_ in littoral sediments and adjacent groundwater can differ by more than 10‰^49^, porewater δ^13^C-CH_4_ has not been shown to differ between canal bottoms and adjacent peat soils^22^. Porewater δ^13^C-CH_4_ collected from 6 profiles (40 to 150 cm depth) located alongside canal waters in our study region had a mean δ^13^C-CH_4_ of −85.0 ± 5.9‰, which was consistently more depleted than any observed canal δ^13^C-CH_4_ value (Supplementary Data 1, Supplementary Data 2). Porewater δ^13^C-CH_4_ varied more between sample depths within each profile than between profiles collected across the landscape, suggesting source δ^13^C-CH_4_ is similarly depleted in ^13^C throughout the study region. Methane production in the water column could also influence canal water CH_4_ concentration and δ^13^C-CH_4_. However, this is unlikely to explain our results because we did not observe net CH_4_ production in any of the laboratory incubations of canal waters, as CH_4_ concentration decreased and δ^13^C-CH_4_ increased in all incubated waters (Fig. 2A, Table S1). If canal water CH_4_ concentration were influenced solely by the total amount of CH_4_ produced and then transported into canal waters, we would expect canal water δ^13^C-CH_4_ to be similarly depleted across canals and not vary systematically with dissolved CH_4_ concentration. Given that CH_4_ concentrations varied ~600-fold alongside a ~40‰ range in δ^13^C-CH_4_, our results indicate that CH_4_ oxidation has a significant influence on canal water CH_4_ concentration and δ^13^C-CH_4_.

As CH_4_ oxidation was a major control of canal water CH_4_ concentration, diffusive CH_4_ emissions were also strongly influenced by the percent of CH_4_ oxidized. Diffusive CH_4_ emissions estimated from dissolved CH_4_ concentration (Supplemental Text 1) ranged from 1.0 to 761.8 mg CH_4_ m^−2^ d^−1^ (mean = 72.2 ± 151.2; median = 18.0) and decreased as the percent of CH_4_ oxidized increased (R^2^ = 0.54, *p* < 0.001; Fig. 3D). Measurements of CH_4_ emissions from floating chamber deployments at a subset of study sites (*n* = 12 canals, mean = 94.9 ± 142.3 CH_4_ m^−2^ d^−1^, median = 33.0, Supplementary Data 1) also indicated a negative relationship between CH_4_ emissions and the percent of CH_4_ oxidized (Fig. S5). By back-calculating what diffusive CH_4_ flux would be in the absence of oxidation, we estimate that CH_4_ oxidation reduces drainage canal CH_4_ emissions by a mean of 136.8 ± 154.1 mg CH_4_ m^−2^ d^−1^ (range: 9.9–684.2). Given the skewed distribution of dissolved CH_4_ concentrations that underlie this estimate, the median value of 72.1 mg CH_4_ m^−2^ d^−1^ (IQR: 36.2–173.6) may be a more robust estimate of the emissions attenuated by CH_4_ oxidation. Overall, our results provide evidence suggesting that CH_4_ oxidation mitigates the majority of potential CH_4_ emissions from canals on the landscape.

### Controls on CH_4_ oxidation in drainage canals

**Fig. 4:**
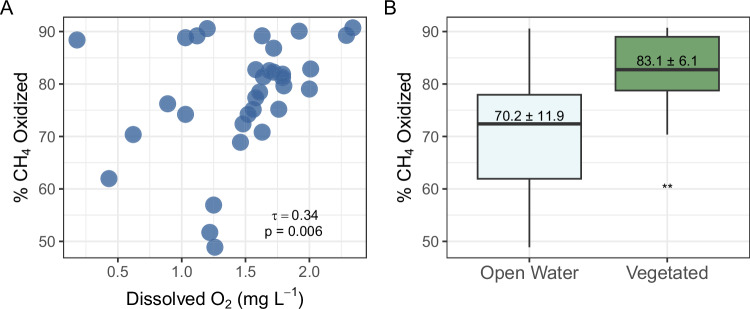
Controls on CH_4_ oxidation in drainage canals. **A** Dissolved oxygen in the surface waters (0–10 cm) of drainage canals versus the percent of CH_4_ oxidized. Each point represents a canal (*n* = 34). **B** Boxplot of the percent of CH_4_ oxidized in open water (light blue, *n* = 19) and vegetated (green, *n* = 15) canals. Within each box the black lines represent median values and the height of the boxes represent the interquartile range. Error bars extend up to 1.5 times the interquartile range. The number in each box represents the mean ± 1 standard deviation of the percent of CH_4_ oxidized for each group. Source data are provided as a Source Data file.

Of the studied controls on CH_4_ oxidation, dissolved oxygen and aquatic vegetation had the most significant influence on the percent of CH_4_ oxidized in canals as determined by canal water δ^13^C-CH_4_. We found that the percent of CH_4_ oxidized increased and dissolved CH_4_ concentration decreased with the concentration of dissolved oxygen at the canal water surface (0-10 cm; *p* < 0.05, Fig. 4A, Table S2). The relationship between dissolved oxygen and CH_4_ oxidation is consistent with oxidation mediated by aerobic methanotrophic bacteria, as has been observed in other stream and river networks^32,46^. While all canals had low dissolved oxygen (0.2 to 2.3 mg L^-1^), methanotrophic bacteria of the order Methylococcales have been shown to have the genetic potential for survival and methanotrophic activity in low oxygen environments^50^. Abundant Methylococcales have been identified in hypoxic tropical freshwaters where paired measurements of dissolved CH_4_ concentration and δ^13^C-CH_4_ indicate ongoing CH_4_ oxidation^34,35^. Our results further support the idea that aerobic CH_4_ oxidation occurs in tropical freshwaters with low dissolved oxygen.

Furthermore, the percent of CH_4_ oxidized was higher in vegetated canals than those with open water (*p* = 0.01, Fig. 4B). Vegetation may enhance CH_4_ oxidation via radial oxygen loss from roots^51,52^ or via oxidation by epiphytic methanotrophs in submersed plants^53^. Although we did not observe a significant difference in dissolved oxygen based on the presence of aquatic vegetation (*p* > 0.05, Table S3), oxygen delivered to the water column by aquatic vegetation is likely rapidly consumed by methanotrophs or by competing aerobic heterotrophs as deposition of more labile organic carbon by aquatic vegetation could stimulate heterotrophic respiration in canal waters^29^. Lower CH_4_ concentrations and more enriched δ^13^C-CH_4_ in vegetated canals could alternatively be explained by plant-mediated emissions^54^, which reduce CH_4_ concentration and enrich the δ^13^C of residual CH_4_ due to the isotopic fractionation of plant-mediated transport^55^. The deposition of labile organic matter from vegetation could also stimulate acetoclastic methanogenesis, which like CH_4_ oxidation would contribute towards larger δ^13^C-CH_4_ in vegetated canals^39^. However, acetoclastic methanogenesis likely contributes little to the δ^13^C-CH_4_ in vegetated canals because hydrogenotrophic methanogenesis has been identified as the dominant pathway in the ombrotrophic tropical peatlands of Southeast Asia^23^ and the Americas^48,56^. Disturbance in peatlands in Southeast Asia has been observed to increase the abundance of plant functional types associated with acetoclastic methanogenesis, like graminoids, but this shift does not appear to increase the abundance of acetoclastic methanogens^57^. While we cannot rule out the possible influence of acetoclastic methanogenesis on canal water δ^13^C-CH_4_, the lower dissolved CH_4_ concentration in vegetated canals (*p* = 0.02, Table S3) lends more support to the idea that vegetation enhances CH_4_ oxidation rather than acetoclastic CH_4_ production in canals.

Given that higher dissolved oxygen and the presence of aquatic vegetation were observed in canals with a shallower water depth (Fig. S6), canal water depth may indirectly mediate CH_4_ oxidation in drainage canal waters. Overall, dissolved oxygen in the surface water of canals (0–10 cm) decreased with the depth of water present in the canal (Kendall’s **τ** = −0.41, *p* < 0.05, Fig. S6). Dissolved CH_4_ concentration, and therefore estimated diffusive emissions, also had a weak but significant positive correlation with canal water depth (**τ** = 0.26, *p* = 0.03, Table S2). This result contradicts previous findings in drainage ditches in temperate peatlands where CH_4_ emissions had a weak negative correlation with depth^58^, but these differing results may be explained by how well canal waters are mixed and aerated. For example, while we observed CH_4_ oxidation in canals where dissolved oxygen is low (<2.5 mg L^−1^) at the surface, dissolved oxygen may become depleted at depth^29,45^ to below the concentration needed for aerobic methanotrophs with high oxygen affinity. As such, CH_4_ oxidation may be limited to the surface waters of deeper canals, while in shallower canals oxidation may occur throughout the water column. Our study also only explicitly considered diffusive emissions. Measurements of CH_4_ ebullition from canals could further clarify the role of water depth in shaping net canal CH_4_ emissions, as ebullitive emissions vary with water depth^59^. Altogether, our results suggest that shallower, vegetated canals may attenuate a higher percentage of CH_4_ emissions through CH_4_ oxidation.

Land use and seasonal precipitation cycles can both influence canal water depth and therefore dissolved oxygen. While we did not observe a significant impact of peatland land use on CH_4_ oxidation nor other parameters including dissolved oxygen (Table S4), peatland water table, which directly influences canal water levels, has been shown to vary significantly between land use types^60^. Canal water depth also varies 2- to 5-fold throughout the year in response to precipitation (Fig. S7), and reduced precipitation and flow during drier months may facilitate oxygen depletion by limiting turbulent mixing and re-aeration of canal waters^27,61^. Accordingly, past studies have reported higher canal CH_4_ emissions during dry periods^27,28^. While our study was not conducted during pronounced wet or dry periods, the dissolved CH_4_ and oxygen concentrations measured in our study fall within the range observed across Southeast Asia under varying land uses and seasons^22,28,45,62,63^ (Table S5). As such, we anticipate that water column CH_4_ oxidation is prevalent across canals draining degraded peatlands in Southeast Asia.

### Influence of oxidation on CH_4_ emissions and their ^13^C in drained tropical peatlands

Our observations of canal CH_4_ emissions estimated from dissolved CH_4_ concentration (72.2 ± 151.2 mg CH_4_ m^-2^ d^-1^) and collected using floating chambers (94.9 ± 142.3 mg CH_4_ m^-2^ d^-1^) are within range of past observations from Indonesia^27,28,64^ and Malaysia^26^ where mean emissions range from 2.8 to 1073 mg CH_4_ m^-2^ d^-1^ (Table S6). The IPCC CH_4_ Emissions Factor for canals in tropical peatlands of 618.9 mg CH_4_ m^-2^ d^-1^ (2259 kg CH_4_ ha^-1^ y^-1^) was based on the only reported data^27^ at the time of the 2013 Wetlands Supplement^65^ This emission factor now represents the high end of field estimates to date among a still small number of existing studies and should be reconsidered to more accurately inventory the anthropogenic (e.g., from land use change) component^66^ of CH_4_ emissions from degraded tropical peatlands.

Despite high oxidation efficiencies, drainage canals can still emit large amounts of CH_4_. For example, in canals where ~50% of the CH_4_ transported from peatlands is oxidized we observe emissions >200 mg CH_4_ m^-2^ d^-1^ (Figs. 3,  S5). The canals in this study were primarily situated in smallholder agricultural systems (Supplementary Data 1), and the mean estimated diffusive CH_4_ emissions from canals presented here are 30x larger on a per area basis than mean peat soil CH_4_ emissions from smallholder agriculture fields in West Kalimantan^67^. Thus, while CH_4_ oxidation plays a critical role in attenuating canal CH_4_ emissions, canals can still contribute significantly to landscape-level CH_4_ emissions from drained peatlands in Southeast Asia.

**Fig. 5:**
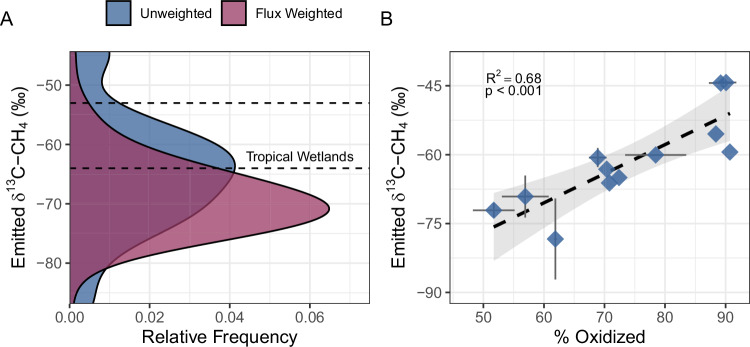
The isotopic composition of CH_4_ emissions from tropical peatland drainage canals. **A** Probability density estimates of the δ^13^C of CH_4_ emitted from canal waters showing the unweighted (blue) and flux-weighted (purple) distributions of emitted δ^13^C-CH_4_. The dashed black lines show the range of δ^13^C of tropical wetland CH_4_ emissions reported in ref. ^68^. **B** The percent of CH_4_ oxidized in drainage canal waters (estimated from dissolved δ^13^C-CH_4_ using α_ox_ = 1.022) versus the δ^13^C of CH_4_ emitted from the corresponding canal. Each point represents a canal (*n* = 12) and error bars show the mean ± 1 standard deviation if replicates were collected at a canal. The shaded regions represent the 95% confidence interval associated with the linear relationship shown in panel B. Source data are provided as a Source Data file.

Beyond the rate of emissions, the δ^13^C signature of CH_4_ emitted from tropical wetlands and freshwaters are critical for constraining their contribution to the global CH_4_ budget, as δ^13^C-CH_4_ values underpin source partitioning by atmospheric inversion models. Using a floating chamber to capture CH_4_ emitted from a subset of the studied canals, we found that the mean δ^13^C-CH_4_ was -64.7 ± 10.5‰ (Fig. 5A). Canal CH_4_ emissions generally decreased as emitted δ^13^C-CH_4_ increased (Fig. S8), therefore the flux-weighted mean δ^13^C-CH_4_ was more negative at -69.0 ± 5.7‰ (Fig. 5A). Past observations of the δ^13^C signature of tropical wetland CH_4_ emissions^68^ indicate a range of -64‰ to -53‰. Our results suggest that the δ^13^C signature of CH_4_ emissions from drainage canals, and potentially drained peatlands in Southeast Asia as whole due to the contribution of canals to landscape-scale CH_4_ emissions, is more negative than prior measurements from tropical wetlands. As such, implementing a distinct δ^13^C-CH_4_ source signature for Southeast Asian peatlands may improve top-down estimates of their CH_4_ emissions.

Furthermore, we find that the variation in δ^13^C of CH_4_ emissions from the canal water surface (−86.9 to -44.3‰) was largely explained by the percent of CH_4_ oxidized in canal waters (R^2^ = 0.68, *p* < 0.05; Fig. 5B). Previous studies have identified oxidation, alongside variation in methanogenic pathways and wetland vegetation, as one potential explanation for latitudinal differences in the δ^13^C of wetland CH_4_ emissions^68–70^. Our results indicate that once CH_4_ produced in peat soils is transported into canals, both the magnitude and the isotopic signature of canal CH_4_ emissions are strongly influenced by CH_4_ oxidation.

In summary, we demonstrate that CH_4_ oxidation can substantially attenuate CH_4_ emissions from canals draining peatlands in Southeast Asia. We estimate that CH_4_ oxidation mitigates >50% of potential CH_4_ emissions from canals across West Kalimantan, Indonesia. As landscape-scale measurements of CH_4_ exchange in drained tropical peatlands indicate that canal networks contribute disproportionately to emissions from these ecosystems^20^, our results suggest that CH_4_ oxidation influences emissions not only from drainage canals but from degraded peatlands in Southeast Asia as a whole. Our results also have implications for peatland CH_4_ emissions in response to land use change, including peatland restoration efforts. For example, we find that oxidation attenuates more CH_4_ emissions from shallower canals that have higher dissolved oxygen concentrations. As such, efforts to rewet drained peatlands in Southeast Asia through canal blocking may impact CH_4_ oxidation and therefore canal CH_4_ emissions through changing canal water depth. Given the extensive networks of drainage canals in Southeast Asia and their substantial contribution to peatland CH_4_ emissions, land use changes impacting CH_4_ oxidation in canals will be reflected in the contribution of peatlands in Southeast Asia to the global CH_4_ budget.

## Methods

### Field sampling

Drainage canals in lowland peatlands were sampled in Kubu Raya and Mempawah Districts, West Kalimantan, Indonesia. Canals were sampled in Kubu Raya in May 2023 and Mempawah in April 2024. This region has an equatorial rainfall pattern with no clear wet and dry season^71^. There is heavy rainfall year-round, but the driest months of the year usually occur in July or August. We sampled waters from canals of different sizes (5 to 90 cm water depths, 0.5 to 6 m canal widths), canals with (*n* = 15) and without aquatic vegetation (*n* = 19), and canals situated on peatlands under a variety of land uses. Smallholder mixed agriculture is the most represented land use in this study, but the sampled canals also include areas in smallholder plantations (pineapple and oil palm), industrial oil palm plantations, and open undeveloped land (i.e., deforested and/or burned areas), as well as 1 canal in a degraded forest, to capture the heterogeneity of drainage canals in the region. At each canal, we measured the canal dimensions as well as water temperature (°C), pH, dissolved oxygen (mg L^-1^), conductivity (μS cm^-1^), and redox potential (Eh, in mV) using a Hanna Instruments HI9829 multiparameter meter. A summary of the canals included in this study is available in Supplementary Data 1.

To measure the isotopic composition of source CH_4_, we collected porewater profiles at 6 locations adjacent to a subset of the sampled canals. As shallow porewater is the primary source of discharge to drainage canals^22^, porewater was collected from 4-5 depths between 40 cm and 150 cm pending water table depth. Porewater was collected using a portable piezometer made of 3/8” stainless steel tubing housing 1/4” polyethylene tubing equipped with a coarse polypropylene screen to prevent collection of coarse debris (SedPoints, M.H.E. Products). Porewater samples were stored in 12 mL glass Exetainer^TM^ vials (Labco Ltd.) without headspace and acidified in the field to a pH of less than 2 using 1.5 M HCl.

### Canal CH_4_ concentration and δ^13^C

We collected surface water samples for analysis of dissolved CH_4_ concentration and δ^13^C-CH_4_ at all canals. Canal water samples were collected approximately 5 cm below the water surface and stored in 12 mL glass Exetainer^TM^ vials (Labco Ltd.) without headspace. Canal waters collected for assessment of in situ dissolved CH_4_ concentration and δ^13^C-CH_4_ were acidified in the field to a pH of less than 2 using 1.5 M HCl. Canal water samples collected in 2023 (including incubations described below) and all porewater samples were analyzed at the Stable Isotope Facility at UC Davis via a Delta V Plus IRMS following headspace equilibration. Samples collected in 2024 were analyzed at Stanford University via a Picarro G2210-*i* cavity ring down spectrometer following headspace equilibration. Reference standards with CH_4_ mixing ratios and δ^13^C-CH_4_ of 10 ppm/-45.5‰ and 30 ppm/-69.0‰ were run before and after sample analysis on the Picarro G2210-*i* to check for accuracy and instrument drift. Dissolved CH_4_ concentrations were calculated considering the mixing ratio of CH_4_ in the equilibrated headspace, using the ideal gas law, and in solution, following Henry’s Law, using the neonDissGas package^72^.

### Incubations

We collected canal waters at a subset (*n* = 13) of the drainage canals for incubation experiments. Surface waters (~5 cm) were collected for all canals included in the incubation experiments, and at 5 of the canals we collected water from ~10 cm above the canal bottom using gas-tight tubing and a hand pump. Collecting these deeper canal waters for incubation experiments enabled us to account for any variability in isotopic fractionation of CH_4_ oxidation with depth in the water column that could impact our estimates of oxidation efficiency. For canal waters collected for incubation experiments, we collected waters as described above but only field acidified samples for the initial incubation time point. Incubations occurred in the dark at room temperature (~25 °C) for 3 days. Duplicate samples for each canal (and depth, if applicable) were acidified every ~24 hours to pH <2 using 1.5 M HCl to stop CH_4_ oxidation. All incubated waters were analyzed at the Stable Isotope Facility at UC Davis. Dissolved CH_4_ concentration was calculated as described above.

Dissolved CH_4_ concentration fell below the limit of quantification within 72 hours, as such incubation results only consider data from the first 2 days. One of the 5 deeper canal waters was omitted as CH_4_ was not detectable after 24 hours. We calculated potential oxidation rates as the change in CH_4_ concentration over the total incubation time. We also calculated the fractionation factor of CH_4_ oxidation, or α_ox_, from the CH_4_ mixing ratios (in ppm) and δ^13^C-CH_4_ of the incubated waters using a simplified Rayleigh model^40^:

1 $${{{\mathrm{ln}}}}\left(\frac{{{{{\rm{CH}}}}}_{4}}{{{{{\rm{CH}}}}}_{4,0}}\right)=\frac{{{{{\rm{\alpha }}}}}_{{{{\rm{ox}}}}}}{1-{{{{\rm{\alpha }}}}}_{{{{\rm{ox}}}}}} * {{{\mathrm{ln}}}}\left(\frac{1000\,+\,{{{{\rm{\delta }}}}}^{13}{{{{\rm{C}}}}-{{{\rm{CH}}}}}_{4}}{1000\,+\,{{{{\rm{\delta }}}}}^{13}{{{{\rm{C}}}}-{{{\rm{CH}}}}}_{4},0}\right)$$

Plotting Eq. (1) with ln(1000 + δ^13^C-CH_4_) on the x-axis and ln(CH_4_) on the y-axis produces a line with a slope of (α_ox_/1-α_ox_). As such, we calculated the slope as the difference in ln(CH_4_) between the initial and final time points over the difference in ln(1000 + δ^13^C-CH_4_) over the same time and then solved for α_ox_.

### Canal CH_4_ emissions

We used a floating chamber to manually collect chamber headspace gasses at 12 of the sampled canals to assess CH_4_ emissions and emitted δ^13^C-CH_4_. A 20 cm diameter/2.1 L floating chamber was deployed on the canal water surfaces for 6 minutes in 2023 and 12 minutes in 2024. Chamber deployment time was increased in 2024 to ensure sufficient CH_4_ accumulation for analysis via Picarro CRDS. The floating chamber was not held in place, but due to low canal water flow (stagnant to ~0.1 m s^-1^) that chamber did not travel during flux measurement. Three 15 mL gas samples were collected from the chamber headspace over the deployment time via a sampling syringe and injected into a pre-evacuated 12 mL glass Exetainer^TM^ vial (Labco Ltd.). Floating chamber headspace gas samples from 2023 were analyzed at the Stable Isotope Facility at UC Davis and from 2024 at Stanford University, as described above. Methane emissions were calculated as the linear increase in chamber headspace CH_4_ mixing ratio over the measurement period and converted from ppm CH_4_ min^-1^ to mg CH_4_ m^-2^ d^-1^ using the Ideal Gas Law and the floating chamber dimensions. Fluxes were accepted if the linear increase in CH_4_ over time met the standards of R^2^ > 0.9 and *p* < 0.05. Emitted δ^13^C-CH_4_ was determined via a Keeling plot approach, in which the δ^13^C of CH_4_ emissions is the y-intercept of a linear regression of the inverse mixing ratio of CH_4_ versus the δ^13^C-CH_4_ of the corresponding sample^73,74^.

We calculated gas transfer velocity (k, m d^-1^) using data from the subset of canals where paired floating chamber CH_4_ fluxes and canal water CH_4_ concentrations were collected using Eq. (2):

2 $${{{\rm{Flux}}}}={{{\rm{k}}}}({{{\rm{C}}}}{{{{\rm{H}}}}}_{4-{{{\rm{canal}}}}}-{{{\rm{C}}}}{{{{\rm{H}}}}}_{4-{{{\rm{eq}}}}})$$

Where CH_4-canal_ is the concentration of CH_4_ in canal water, CH_4-eq_ is the CH_4_ concentration at equilibrium the atmosphere (CH_4-eq_), and flux is the rate of CH_4_ emissions measured using the floating chamber. We used the median k value from the floating chamber deployments to estimate diffusive fluxes across all sampled (*n* = 34) canals. While applying a uniform value introduces uncertainty into the estimates of diffusive fluxes, conditions across the study region are characterized by high canal water temperature, low canal flow velocity (~0.1 m s^-1^), and low windspeed. As such, factors that strongly influence CH_4_ degassing (e.g., solubility and turbulence) should have minimal variation relative to the ~600-fold variation in canal water CH_4_ concentration across study sites. Values were normalized to k_600_ for literature comparison. See Supplementary Text 1 for further discussion of approaches to estimate k.

### Estimating percent oxidation

We used a simple box model to estimate the percent of CH_4_ transported from drained peatlands into canals that is oxidized and therefore not emitted to the atmosphere. The model calculated the percent oxidized based on the difference in δ^13^C between the source CH_4_ (e.g., peat porewater) and CH_4_ after oxidation (e.g., in the canal waters) as well as the isotopic fractionation of CH_4_ oxidation (α_ox_), which we determined via incubations as described above. Oxidation efficiency (*f*_ox_) was calculated using a Rayleigh model for closed systems^42,75^:

3 $${{\mathrm{ln}}}(1-{{{{\rm{f}}}}}_{{{{\rm{ox}}}}})=[{{\mathrm{ln}}}({{{{\rm{\delta }}}}}_{{{{\rm{source}}}}}+1000)-{{\mathrm{ln}}}({{{{\rm{\delta }}}}}_{{{{\rm{canal}}}}}+1000)]/[{{{{\rm{\alpha }}}}}_{{{{\rm{ox}}}}}-1]$$

Where δ_source_ and δ_canal_ are the δ^13^C-CH_4_ of peat porewater and drainage canal waters, respectively, and *f*_ox_ is the fraction of CH_4_ oxidized. Values of *f*_ox_ were multiplied by 100 to convert to the percent of CH_4_ oxidized. The closed system approach represents a lower bound on oxidation, as open system models often result in estimates of the percent oxidized >100%.

The results presented in the main analyses and figures are estimates of the percent oxidized based on mean observed values of α_ox_ (1.022 ± 0.009, from incubations) and δ_source_ (-85.0 ± 5.9‰, *n* = 27 measurements from 6 porewater profiles, Supplementary Data 2). To characterize the uncertainty of our estimates due to variability in α_ox_ and δ_source_, we also report how our estimate varies when using ± 1 standard deviation of α_ox_ or δ_source_ in Eq. (3). Varying α_ox_ or δ_source_ by ± 1 standard deviation changes our estimate of the mean percent oxidized by ~10%.

To estimate the amount of CH_4_ emissions attenuated by CH_4_ oxidation, we back-calculated the concentration of CH_4_ in canal waters based on the *f*_ox_ value for each canal:

4 $${{{\rm{Predicted}}}}\;\,{{{\rm{C}}}}{{{{\rm{H}}}}}_{4}\;{{{\rm{Concentration}}}}=\frac{{{{\rm{Observed}}}}\;\,{{{\rm{C}}}}{{{{\rm{H}}}}}_{4}\;{{{\rm{Concentration}}}}}{1\,-\,{{{{\rm{f}}}}}_{{{{\rm{ox}}}}}}$$

Using this predicted concentration, we calculated predicted diffusive CH_4_ fluxes as described above. We then subtracted the diffusive CH_4_ fluxes calculated from observed CH_4_ concentrations from the predicted CH_4_ fluxes based on the back-calculated concentrations to estimate the CH_4_ emissions mitigated by CH_4_ oxidation.

### Statistical analysis

Statistical analysis and data visualization were performed in R v4.0.3. Data preparation was conducted using the dplyr package^76^. Data and analyses were visualized using the ggplot2^77^ and patchwork^78^ packages. Statistical analyses were performed using the R Core Team stats package. Dissolved CH_4_ concentration and estimated diffusive emissions were log_10_-transformed prior to all statistical analysis to improve normality. If there were replicate measurements taken in a canal, the mean value of replicates was used in statistical analysis and data visualization. Summary statistics were calculated using all observations, including spatial replicates, to report the full range of observations. The level of significance for all analyses was 0.05.

We tested relationships between canal water CH_4_ concentration and δ^13^C-CH_4_ and between estimated diffusive CH_4_ fluxes and the percent of CH_4_ oxidized using least squares regression. We used Kendall’s rank correlation to assess the strength and direction of monotonic relationships between dissolved oxygen or canal depth and canal water dissolved CH_4_ concentration, δ^13^C-CH_4_, and percent CH_4_ oxidized. We used a non-parametric correlation for these analyses as relationships between CH_4_ oxidation and dissolved oxygen are often non-linear due to substrate saturation and potential inhibitory effects of oxygen above the optimal levels for CH_4_ oxidation in freshwater environments^79^. We used one-way ANOVA to assess the impact of vegetation and land use on canal properties and CH_4_ cycling.

## Supplementary information

Supplementary Information

Description of Additional Supplementary Files

Supplementary Data 1

Supplementary Data 2

Transparent Peer Review file

## Source data

Source Data

## Data Availability

All data are presented in the manuscript and/or the Supplementary Information. The data used in this study are available at the Zenodo repository under ‘Tropical Peatland Drainage Canal Methane Concentrations, Fluxes, and Isotopic Composition’ (10.5281/zenodo.11155160). Source data are provided with this paper.

## References

[1] Saunois, M. et al. The Global Methane Budget 2000–2017. *Earth Syst. Sci. Data* **12**, 1561–1623 (2020).

[2] Johnson, M. S., Matthews, E., Du, J., Genovese, V. & Bastviken, D. Methane emission from global lakes: new spatiotemporal data and observation‐driven modeling of methane dynamics indicates lower emissions. *J. Geophys. Res. Biogeosci.* **127**, e2022JG006793 (2022). 36250198 10.1029/2022JG006793 PMC9540782

[3] Stavert, A. R. et al. Regional trends and drivers of the global methane budget. *Glob. Change Biol.* **28**, 182–200 (2022). 10.1111/gcb.15901 PMC9298116 34553464

[4] Rocher-Ros, G. et al. Global methane emissions from rivers and streams. *Nature* **621**, 530–535 (2023). 37587344 10.1038/s41586-023-06344-6 PMC10511311

[5] Feng, L., Palmer, P. I., Zhu, S., Parker, R. J. & Liu, Y. Tropical methane emissions explain large fraction of recent changes in global atmospheric methane growth rate. *Nat. Commun.* **13**, 1378 (2022). 35297408 10.1038/s41467-022-28989-z PMC8927109

[6] Nisbet, E. G. et al. Rising atmospheric methane: 2007–2014 growth and isotopic shift. *Glob. Biogeochem. Cycles* **30**, 1356–1370 (2016).

[7] Yin, Y. et al. Accelerating methane growth rate from 2010 to 2017: leading contributions from the tropics and East Asia. *Atmos. Chem. Phys.* **21**, 12631–12647 (2021).

[8] Qu, Z. et al. Inverse modeling of 2010–2022 satellite observations shows that inundation of the wet tropics drove the 2020–2022 methane surge. *Proc. Natl Acad. Sci.* **121**, e2402730121 (2024). 39316054 10.1073/pnas.2402730121 PMC11459126

[9] Dhandapani, S. & Evers, S. Oil palm ‘slash-and-burn’ practice increases post-fire greenhouse gas emissions and nutrient concentrations in burnt regions of an agricultural tropical peatland. *Sci. Total Environ.* **742**, 140648 (2020). 32721749 10.1016/j.scitotenv.2020.140648

[10] Hergoualc’h, K. et al. Spatial and temporal variability of soil N_2_O and CH_4_ fluxes along a degradation gradient in a palm swamp peat forest in the Peruvian Amazon. *Glob. Change Biol.* **26**, 7198–7216 (2020). 10.1111/gcb.15354 PMC7756671 32949077

[11] Swails, E., Frolking, S., Deng, J. & Hergoualc’h, K. Degradation increases peat greenhouse gas emissions in undrained tropical peat swamp forests. *Biogeochemistry* **167**, 59–74 (2024).

[12] Darusman, T., Murdiyarso, D., Impron & Anas, I. Effect of rewetting degraded peatlands on carbon fluxes: a meta-analysis. *Mitig. Adapt. Strateg. Glob. Change* **28**, 10 (2023).

[13] Novita, N. et al. Strong climate mitigation potential of rewetting oil palm plantations on tropical peatlands. *Sci. Total Environ.* **952**, 175829 (2024). 39197784 10.1016/j.scitotenv.2024.175829

[14] Watanabe, A., Purwanto, B. H., Ando, H., Kakuda, K. & Jong, F.-S. Methane and CO2 fluxes from an Indonesian peatland used for sago palm (Metroxylon sagu Rottb.) cultivation: Effects of fertilizer and groundwater level management. *Agric. Ecosyst. Environ.* **134**, 14–18 (2009).

[15] Hoyt, A. M., Chaussard, E., Seppalainen, S. S. & Harvey, C. F. Widespread subsidence and carbon emissions across Southeast Asian peatlands. *Nat. Geosci.* **13**, 435–440 (2020).

[16] Miettinen, J., Hooijer, A., Vernimmen, R., Liew, S. C. & Page, S. E. From carbon sink to carbon source: extensive peat oxidation in insular Southeast Asia since 1990. *Environ. Res. Lett.* **12**, 024014 (2017).

[17] Dadap, N. C. et al. Drainage canals in Southeast Asian Peatlands increase carbon emissions. *AGU Adv.* **2**, e2020AV000321 (2021).

[18] Miettinen, J., Shi, C. & Liew, S. C. Land cover distribution in the peatlands of Peninsular Malaysia, Sumatra and Borneo in 2015 with changes since 1990. *Glob. Ecol. Conserv.* **6**, 67–78 (2016).

[19] Cooper, H. V. et al. Greenhouse gas emissions resulting from conversion of peat swamp forest to oil palm plantation. *Nat. Commun.* **11**, 407 (2020). 31964892 10.1038/s41467-020-14298-w PMC6972824

[20] Deshmukh, C. S. et al. Impact of forest plantation on methane emissions from tropical peatland. *Glob. Change Biol.* **26**, 2477–2495 (2020). 10.1111/gcb.15019 PMC7155032 31991028

[21] Dhandapani, S., Ritz, K., Evers, S., Yule, C. M. & Sjögersten, S. Are secondary forests second-rate? Comparing peatland greenhouse gas emissions, chemical and microbial community properties between primary and secondary forests in Peninsular Malaysia. *Sci. Total Environ.* **655**, 220–231 (2019). 30471590 10.1016/j.scitotenv.2018.11.046

[22] Somers, L. D. et al. Processes Controlling Methane Emissions From a Tropical Peatland Drainage Canal. *J. Geophys. Res. Biogeosci.* **128**, e2022JG007194 (2023).

[23] Hoyt, A. M. Carbon Fluxes from Tropical Peatlands: Methane, Carbon Dioxide, and Peatland Subsidence. (Massachusetts Institute of Technology, 2017).

[24] Peacock, M. et al. Global importance of methane emissions from drainage ditches and canals. *Environ. Res. Lett.* **16**, 044010 (2021).

[25] Roulet, N. T. & Moore, T. R. The effect of forestry draining practices on the emission of methane from northern peatlands. *Can. J. Res.* **25**, 491–499 (1995).

[26] Manning, F. C., Kho, L. K., Hill, T. C., Cornulier, T. & Teh, Y. A. Carbon emissions from oil palm plantations on peat soil. *Front. Glob. Change* **2**, 37 (2019).

[27] Jauhiainen, J. & Silvennoinen, H. Diffusion GHG fluxes at tropical peatland drainage canal water surfaces. *Suoseura* **63**, 93–105 (2012).

[28] Kent, M. S. Greenhouse gas emissions from channels draining intact and degraded tropical peat swamp forest. (The Open University, 2019).

[29] Bowen, J. C., Wahyudio, P. J., Anshari, G. Z., Aluwihare, L. I. & Hoyt, A. M. Canal networks regulate aquatic losses of carbon from degraded tropical peatlands. *Nat. Geosci.* **17**, 213–218 (2024).

[30] Barbosa, P. M. et al. High rates of methane oxidation in an Amazon floodplain lake. *Biogeochemistry* **137**, 351–365 (2018).

[31] Guérin, F. & Abril, G. Significance of pelagic aerobic methane oxidation in the methane and carbon budget of a tropical reservoir. *J. Geophys. Res. Biogeosci.* **112**, 2006JG000393 (2007).

[32] Sawakuchi, H. O. et al. Oxidative mitigation of aquatic methane emissions in large Amazonian rivers. *Glob. Change Biol.* **22**, 1075–1085 (2016). 10.1111/gcb.13169 26872424

[33] Pierangeli, G. M. F. et al. Higher abundance of sediment methanogens and methanotrophs do not predict the atmospheric methane and carbon dioxide flows in eutrophic tropical freshwater reservoirs. *Front. Microbiol.* **12**, 647921 (2021). 33815337 10.3389/fmicb.2021.647921 PMC8010658

[34] Reis, P. C. J., Ruiz-González, C., Crevecoeur, S., Soued, C. & Prairie, Y. T. Rapid shifts in methanotrophic bacterial communities mitigate methane emissions from a tropical hydropower reservoir and its downstream river. *Sci. Total Environ.* **748**, 141374 (2020). 32823225 10.1016/j.scitotenv.2020.141374

[35] Zigah, P. K. et al. Methane oxidation pathways and associated methanotrophic communities in the water column of a tropical lake: Lake Kivu methane oxidation pathways. *Limnol. Oceanogr.* **60**, 553–572 (2015).

[36] Moore, S. et al. Deep instability of deforested tropical peatlands revealed by fluvial organic carbon fluxes. *Nature* **493**, 660–663 (2013). 23364745 10.1038/nature11818

[37] Yupi, H. M., Inoue, T. & Bathgate, J. Concentrations, loads and yields of organic carbon from two tropical peat swamp forest streams in Riau Province, Sumatra, Indonesia. *Mires Peat* 1–15 (2016) 10.19189/MaP.2015.OMB.181.

[38] Coleman, D. D., Risatti, J. B. & Schoell, M. Fractionation of carbon and hydrogen isotopes by methane-oxidizing bacteria. *Geochim. Cosmochim. Acta* **45**, 1033–1037 (1981).

[39] Whiticar, M. J. Carbon and hydrogen isotope systematics of bacterial formation and oxidation of methane. *Chem. Geol.* **161**, 291–314 (1999).

[40] Mahieu, K., Visscher, A. D., Vanrolleghem, P. A. & Cleemput, O. V. Carbon and hydrogen isotope fractionation by microbial methane oxidation: Improved determination. *Waste Manag* **26**, 389–398 (2006). 16442790 10.1016/j.wasman.2005.11.006

[41] Liptay, K., Chanton, J., Czepiel, P. & Mosher, B. Use of stable isotopes to determine methane oxidation in landfill cover soils. *J. Geophys. Res. Atmos.* **103**, 8243–8250 (1998).

[42] Bastviken, D., Ejlertsson, J. & Tranvik, L. Measurement of methane oxidation in lakes: a comparison of methods. *Environ. Sci. Technol.* **36**, 3354–3361 (2002). 12188365 10.1021/es010311p

[43] Thottathil, S. D., Reis, P. C. J. & Prairie, Y. T. Variability and controls of stable carbon isotopic fractionation during aerobic methane oxidation in temperate lakes. *Front. Environ. Sci.* **10**, 833688 (2022).

[44] Zhang, G. B. et al. Pathway of CH_4_ production, fraction of CH_4_ oxidized, and ^13^C isotope fractionation in a straw-incorporated rice field. *Biogeosciences* **10**, 3375–3389 (2013).

[45] Gandois, L. et al. From canals to the coast: dissolved organic matter and trace metal composition in rivers draining degraded tropical peatlands in Indonesia. *Biogeosciences* **17**, 1897–1909 (2020).

[46] Robison, A. L. et al. Dominance of diffusive methane emissions from lowland headwater streams promotes oxidation and isotopic enrichment. *Front. Environ. Sci.* **9**, 791305 (2022).

[47] Taillardat, P. et al. Carbon dioxide and methane dynamics in a peatland headwater stream: origins, processes and implications. *J. Geophys. Res. Biogeosci.* **127**, e2022JG006855 (2022).

[48] Holmes, M. E., Chanton, J. P., Tfaily, M. M. & Ogram, A. CO_2_ and CH_4_ isotope compositions and production pathways in a tropical peatland. *Glob. Biogeochem. Cycles* **29**, 1–18 (2015).

[49] Schenk, J. et al. Methane in lakes: variability in stable carbon isotopic composition and the potential importance of groundwater input. *Front. Earth Sci.* **9**, 722215 (2021).

[50] Reis, P. C. J. et al. Enigmatic persistence of aerobic methanotrophs in oxygen-limiting freshwater habitats. *ISME J.* **18**, wrae041 (2024). 38470309 10.1093/ismejo/wrae041 PMC11008690

[51] Girkin, N. T., Vane, C. H., Turner, B. L., Ostle, N. J. & Sjögersten, S. Root oxygen mitigates methane fluxes in tropical peatlands. *Environ. Res. Lett.* **15**, 064013 (2020).

[52] Määttä, T. & Malhotra, A. The hidden roots of wetland methane emissions. *Glob. Change Biol.* **30**, e17127 (2024). 10.1111/gcb.17127 38337165

[53] Heilman, M. A. & Carlton, R. G. Methane oxidation associated with submersed vascular macrophytes and its impact on plant diffusive methane flux. *Biogeochemistry* **52**, 207–224 (2001).

[54] Akhtar, H. et al. Significant sedge-mediated methane emissions from degraded tropical peatlands. *Environ. Res. Lett*. (2021) 10.1088/1748-9326/abc7dc.

[55] Chanton, J. P. The effect of gas transport on the isotope signature of methane in wetlands. *Org. Geochem.* **36**, 753–768 (2005).

[56] Buessecker, S. et al. Microbial communities and interactions of nitrogen oxides with methanogenesis in diverse peatlands of the Amazon basin. *Front. Microbiol.* **12**, 659079 (2021). 34267733 10.3389/fmicb.2021.659079 PMC8276178

[57] Bandla, A., Akhtar, H., Lupascu, M., Sukri, R. S. & Swarup, S. Elevated methane flux in a tropical peatland post-fire is linked to depth-dependent changes in peat microbiome assembly. *Npj Biofilms Microbiomes* **10**, 8 (2024). 38253600 10.1038/s41522-024-00478-9 PMC10803758

[58] Vermaat, J. E. et al. Greenhouse gas fluxes from Dutch peatland water bodies: importance of the surrounding landscape. *Wetlands* **31**, 493–498 (2011).

[59] Sawakuchi, H. O. et al. Methane emissions from Amazonian Rivers and their contribution to the global methane budget. *Glob. Change Biol.* **20**, 2829–2840 (2014). 10.1111/gcb.12646 24890429

[60] Hirano, T., Kusin, K., Limin, S. & Osaki, M. Evapotranspiration of tropical peat swamp forests. *Glob. Change Biol.* **21**, 1914–1927 (2015). 10.1111/gcb.12653 24912043

[61] Liu, M. et al. Effects of rainfall on thermal stratification and dissolved oxygen in a deep drinking water reservoir. *Hydrol. Process.* **34**, 3387–3399 (2020).

[62] Waldron, S. et al. C mobilisation in disturbed tropical peat swamps: old DOC can fuel the fluvial efflux of old carbon dioxide, but site recovery can occur. *Sci. Rep.* **9**, 11429 (2019). 31391485 10.1038/s41598-019-46534-9 PMC6685963

[63] Thornton, S. A., Dudin,., Page, S. E., Upton, C. & Harrison, M. E. Peatland fish of Sebangau, Borneo: diversity, monitoring and conservation. *Mires Peat.* 1–25 (2018) 10.19189/MaP.2017.OMB.313.

[64] Swails, E. et al. Soil nitrous oxide and methane fluxes from a land-use change transition of primary forest to oil palm in an Indonesian peatland. *Biogeochemistry* **167**, 363–381 (2023).

[65] Hiraishi, T. et al. *2013 Supplement to the 2006 IPCC Guidelines for National Greenhouse Gas Inventories: Wetlands: Methodological Guidance on Lands with Wet and Drained Soils, and Constructed Wetlands for Wastewater Treatment*. (IPCC, Intergovernmental Panel on Climate Change, Hayama, Japan, 2014).

[66] Jackson, R. B. et al. Human activities now fuel two-thirds of global methane emissions. *Environ. Res. Lett.* **19**, 101002 (2024).

[67] Jovani‐Sancho, A. J. et al. CH_4_ and N_2_O emissions from smallholder agricultural systems on tropical peatlands in Southeast Asia. *Glob. Change Biol.* **29**, 4279–4297 (2023). 10.1111/gcb.16747 PMC10946781 37100767

[68] Brownlow, R. et al. Isotopic ratios of tropical methane emissions by atmospheric measurement. *Glob. Biogeochem. Cycles* **31**, 1408–1419 (2017).

[69] Feinberg, A. I., Coulon, A., Stenke, A., Schwietzke, S. & Peter, T. Isotopic source signatures: Impact of regional variability on the δ 13 CH 4 trend and spatial distribution. *Atmos. Environ.* **174**, 99–111 (2018).

[70] Oh, Y. et al. Improved global wetland carbon isotopic signatures support post-2006 microbial methane emission increase. *Commun. Earth Environ.* **3**, 159 (2022).

[71] Aldrian, E. & Dwi Susanto, R. Identification of three dominant rainfall regions within Indonesia and their relationship to sea surface temperature. *Int. J. Climatol.* **23**, 1435–1452 (2003).

[72] Cawley, K., Goodman, K., Weintraub, S. & Parker, S. Neon user guide to dissolved gases in surface water (DP1.20097.001). neonDissGas package. (2020).

[73] Keeling, C. D. The concentration and isotopic abundances of atmospheric carbon dioxide in rural areas. *Geochim. Cosmochim. Acta* **13**, 322–334 (1958).

[74] Pataki, D. E. et al. The application and interpretation of Keeling plots in terrestrial carbon cycle research. *Glob. Biogeochem. Cycles* **17**, 2001GB001850 (2003).

[75] Happell, J. D., Chanton, J. & Showers, W. S. The influence of methane oxidation on the stable isotopic composition of methane emitted from Florida swamp forests. *Geochim. Cosmochim. Acta* **58**, 4377–4388 (1994).

[76] Wickham, H., Francois, R., Henry, L. & Muller, K. dplyr: a grammar of data manipulation. (2021).

[77] Wickham, H. *Ggplot2: Elegant Graphics for Data Analysis*. (Springer-Verlag, New York, 2016).

[78] Pedersen, T. L. Patchwork: the composer of plots. (2020).

[79] Thottathil, S. D., Reis, P. C. J. & Prairie, Y. T. Methane oxidation kinetics in northern freshwater lakes. *Biogeochemistry* **143**, 105–116 (2019).

