# Supplementary material for: Fate of methane in canals draining tropical peatlands
Source: Nat Commun. 2024 Nov 11;15:9766. doi: 10.1038/s41467-024-54063-x (PMC11555057; doi:10.1038/s41467-024-54063-x)
Supplement: Supplementary file 1 — Supplementary Information [file 41467_2024_54063_MOESM1_ESM.pdf]

Supplementary Information for

Fate of methane in canals draining tropical peatlands

Clarice R. Perryman[1]*, Jennifer C. Bowen[1], Julie Shahan[1], Desi Silviani P.A.B.[2], Erin Dayanti[3], Yulita Andriyani[2], Adibtya Asyhari[4], Adi Gangga[4], Nisa Novita[4,5], Gusti Z. Anshari[2,3], Alison M. Hoyt[1]

[1]Department of Earth System Science, Stanford University, Stanford, CA, USA
[2]Department of Soil Science, Tanjungpura University, Pontianak, Indonesia
[3]Magister of Environmental Science, Tanjungpura University, Pontianak, Indonesia
[4]Yayasan Konservasi Alam Nusantara, Jakarta, Indonesia
[5]The Nature Conservancy, Jakarta, Indonesia

Corresponding author: Clarice R. Perryman
Email: crperry@stanford.edu

This PDF file includes:

Supplementary Text 1
Figures S1 to S8
Tables S1 to S6
Supplementary References

Supplementary Text 1

Approaches to estimate diffusive CH_4 fluxes:

Estimates of diffusive CH_4 fluxes from freshwaters depend on the concentration of dissolved CH_4 ($CH_{4\text{-dissolved}}$) and the gas transfer (or piston) velocity (k, Eqn. 1).

$$Flux = k(CH_{4-dissvolved} - CH_{4-eq}) \quad \text{(Eqn. 1)}$$

In the main text, we report estimates of diffusive CH4 flux using a gas exchange velocity determined through manual chamber fluxes at a subset of study sites (n = 12 canals). Another approach is to model k from wind speed using the turbulent boundary layer method, in which k_{600} is the gas exchange coefficient after Cole and Caraco (1998) that is a function of windspeed (U_{10})

$$k_{600} = 2.07 + 0.215 * U_{10}^{1.7} \quad \text{(Eqn. 2)}$$

k_{600} values can then be converted to k values for CH_4 at ambient temperatures using temperature-specific coefficients and Schmidt numbers (S_c).

$$k = k_{600}(S_c/600)^{-0.5} \quad \text{(Eqn. 3)}$$

To compare the impact of the selected approach to estimating the gas exchange velocity, we also modelled k from daily average wind speed from two weather stations in our study region (data from Badan Meteorologi Klimatologi dan Geofisika; https://www.bmkg.go.id/). Modelled k values are lower than those observed from chamber deployments, resulting in lower estimates of diffusive fluxes. The modelled k_{600} values are comparable to those determined in canals in Central Kalimantan by Kent (2019) and other forested shallow water bodies (Holgerson et al., 2017), while the k_{600} values from chamber deployments are in better agreement with observations from northern peatland streams (Taillardat et al., 2022).

**Reference**	**Environment**	**k_{600} (m d^{-1})**
This Study	Drainage Canals – degraded (from chamber deployments)	1.70 ± 1.75 [median: 1.15]
This Study	Drainage Canals – degraded (wind speed method)	0.65 ± 0.16 [median: 0.66]
Kent (2019)	Drainage Canals - forested	0.76
Kent (2019)	Drainage Canals - degraded	0.80
Taillardat et al. (2022)	Peatland headwater stream	0.9-1.7
Taillardat et al. (2022)	Peatland headwater stream – minerotrophic segment	2.4-5.2
Holgerson et al. (2017)	Small forested ponds - daytime	0.19-0.72

Using the modelled k values results in a mean diffusive flux across canals of 38.7 ± 79.5 mg CH_4 m^{-2} d^{-1}. Diffusive fluxes estimated using the chamber-derived gas transfer velocity are in better agreement with the subset of observations made using manual deployments (72.2 ± 151.2 vs. 94.9 ± 142.3 mg CH_4 m^{-2} d^{-1}), as well as other observations from canal draining peatlands in other regions of Southeast Asia (Table S8).

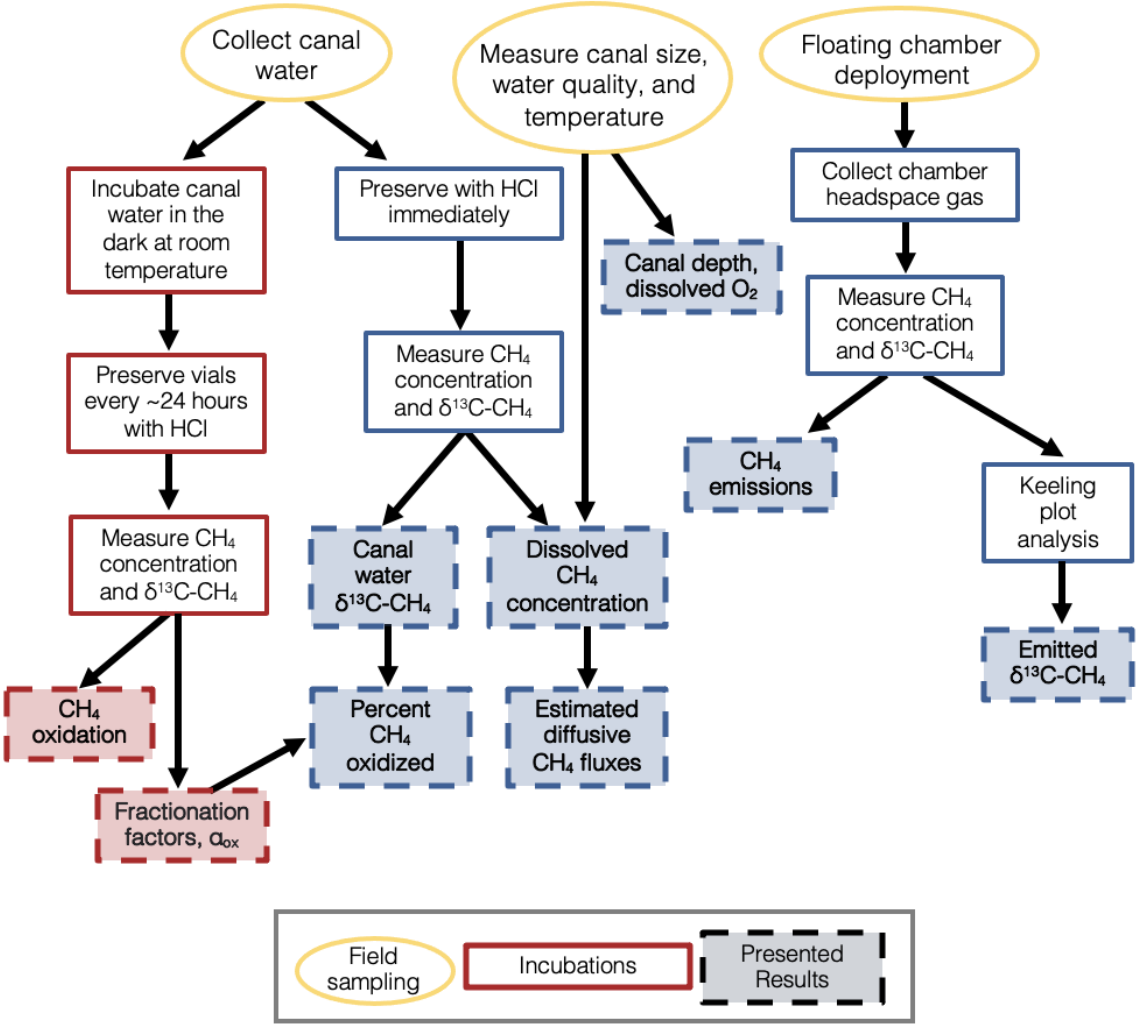

Figure S1. Flow chart outlining methods and workflow for key results from this study.

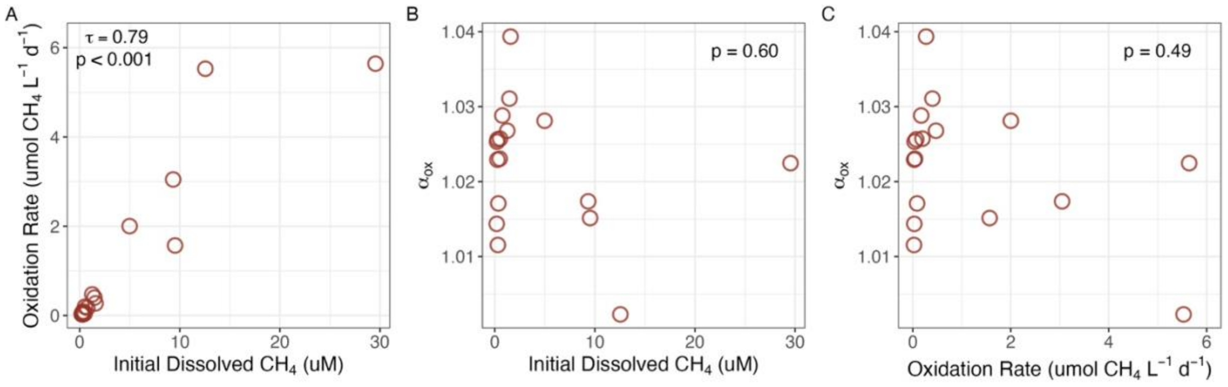

Figure S2. A. CH_4 oxidation rates from incubations of canal waters varied with initial CH_4 concentration. B-C. Isotopic fractionation (α_{ox}) did not vary with initial CH_4 concentration nor CH_4 oxidation rate.

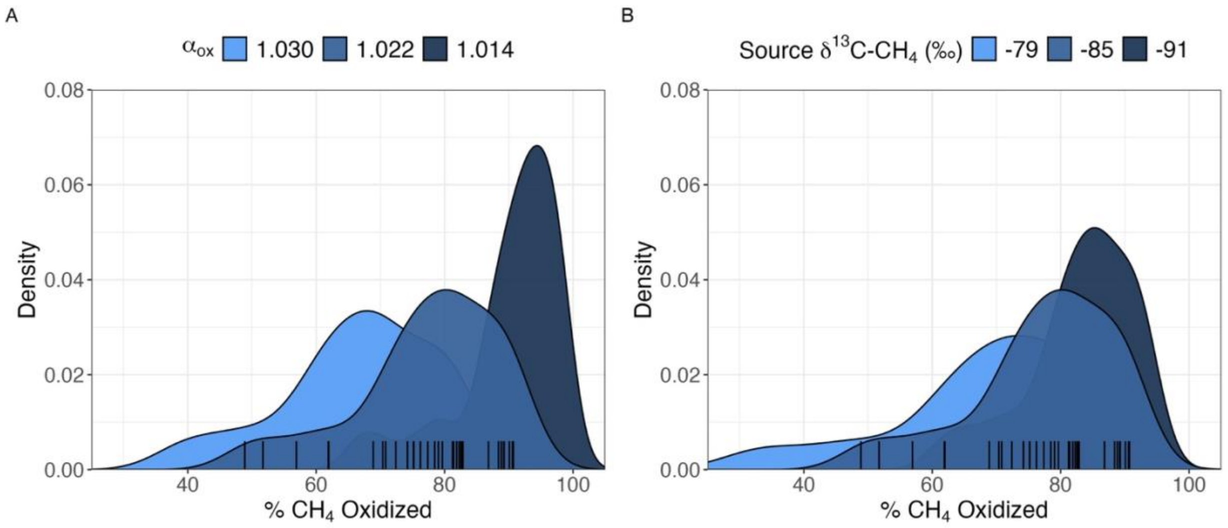

Figure S3. A. Density plot showing estimates of the percent of CH_4 oxidized in canal waters using the mean or ± one standard deviation value of our estimate of the isotopic fractionation of oxidation (1.022 ± 0.008). B. Density plot showing estimates of the percent of CH_4 oxidized in canal waters using the mean or ± one standard deviation value of our estimate of the source $\delta^{13}C$-CH_4 (85.0 ± 5.9‰).

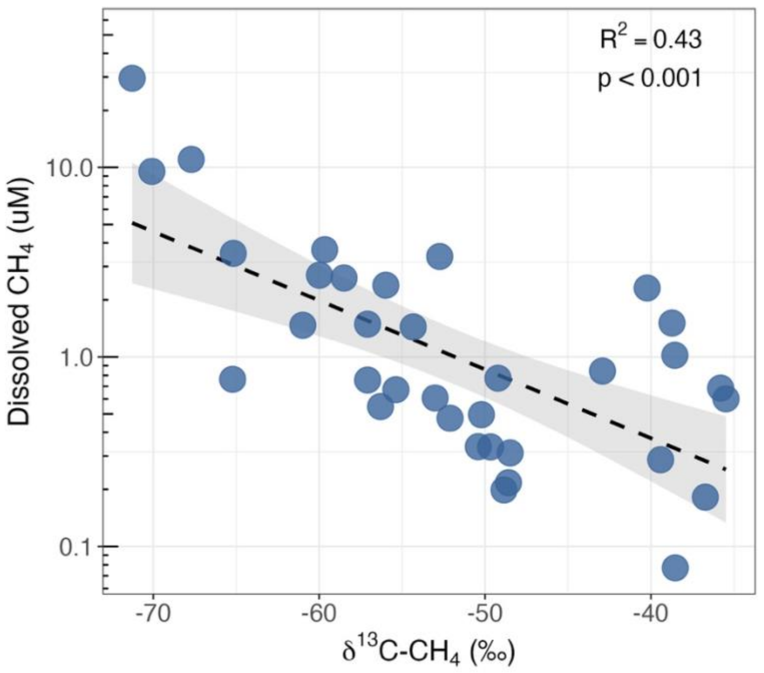

Figure S4. Canal water $\delta^{13}C$-CH_4 and dissolved CH_4 concentration across the studied canals (n = 34). Each dot represents a canal. The shaded region represent the 95% confidence interval associated with the linear relationship. Dissolved CH_4 concentration is shown on a log_{10} scale.

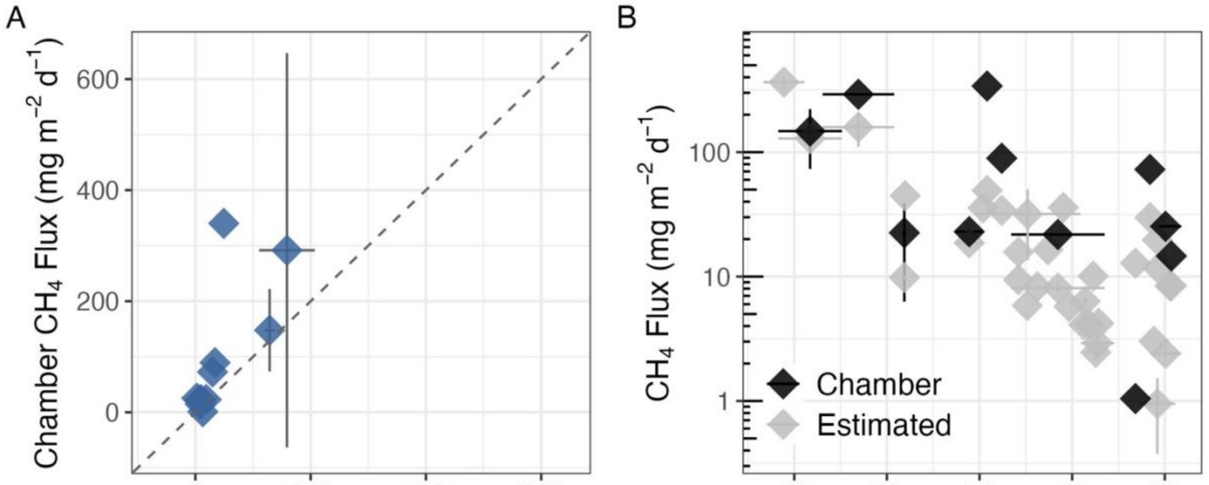

Figure S5. A. Comparison of CH_4 emissions estimated from canal water CH_4 concentrations or directly measured using floating chambers. Dashed line shows 1:1 line. B. Relationship between CH_4 fluxes measured via different methods and the percent of CH_4 oxidized in canal waters. For both panels, each point represents a canal and error bars show the mean ± 1 standard deviation if replicates were collected at a canal.

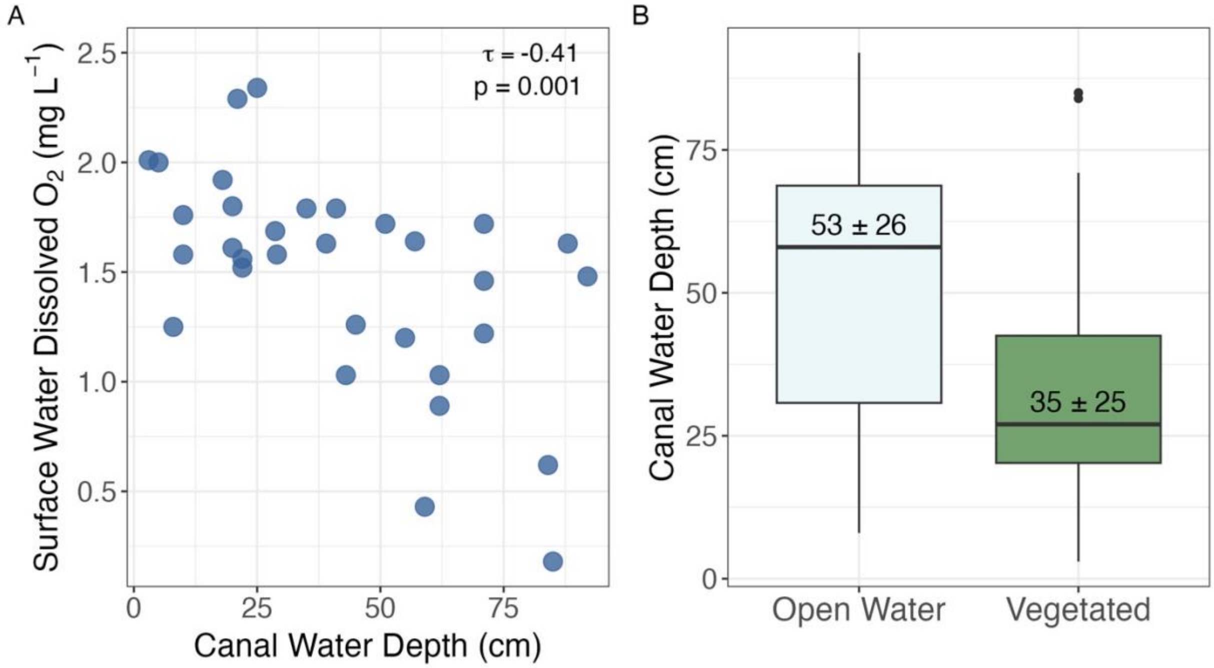

Figure S6. A. Surface water dissolved oxygen decreases as the depth of water in the canal increases. Each point represents a canal (n = 34). B. Boxplot of water depth across canals with (green, n = 15) and without vegetation (light blue, n = 19, ANOVA p = 0.04). Within each box the black lines represent median values and the height of the boxes represent the interquartile range. Error bars extend up to 1.5 times the interquartile range, and black points represent outlier values greater/less than the interquartile range. The number in each box represents the mean ± 1 standard deviation of canal water depth for each group.

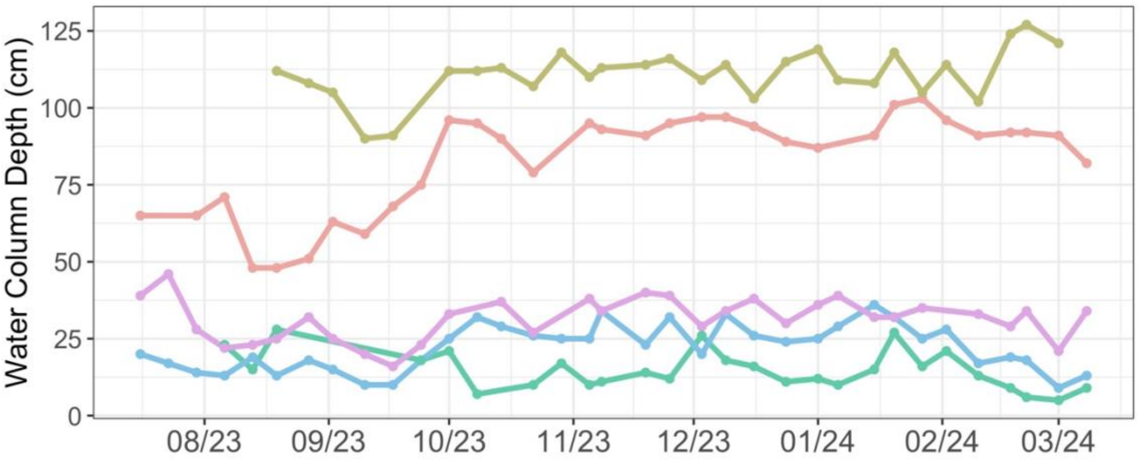

Figure S7. Depth of water present in five of the study canals from July 2023 to March 2024.

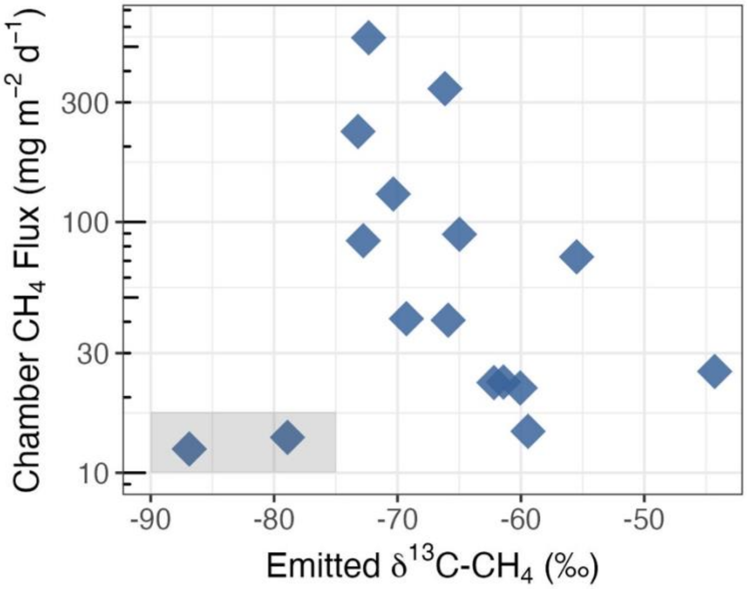

Figure S8. $\delta^{13}C$ of CH_4 emissions captured from a subset of canals using floating chambers versus the corresponding CH_4 flux from each measurement. Grey box indicates two measurements collected near a canal blocking structure which break from the overall negative trend between CH_4 emissions and $\delta^{13}C$-CH_4. Each point represents an individual flux measurement. Methane fluxes on the y-axis are shown on a log_{10} scale.

Table S1. Dissolved CH_4 concentration and $\delta^{13}C$-CH_4 across incubated canal waters. Values are mean ± standard error of 2 replicate vials for each time point. Time indicates the incubation length before determination of the final CH_4 concentrations and $\delta^{13}C$-CH_4. For depth, S = surface and D = deep. Values for the CH_4 concentrations and $\delta^{13}C$-CH_4 of individual replicates are available in the manuscript Source Data file.

Canal	Depth	CH_4 T_0 (μM)	CH_4 T_{final} (μM)	^{13}C T_0 (‰)	^{13}C T_{final} (‰)	Time (hours)
1	S	9.53 ± 0.48	6.02 ± 1.37	-71.1 ± 1.0	-63.7 ± 4.7	53.6
1	D	9.34 ± 0.77	2.17 ± 1.52	-70.5 ± 0.3	-47.1 ± 7.3	56.4
10	S	0.50 ± 0.03	0.39 ± 0.02	-50.2 ± 0.3	-45.3 ± 2.6	53.6
29	S	12.55 ± 0.85	0.04 ± 0.001	-69.0 ± 0.02	-56.8 ± 0.6	54.3
30	S	1.47 ± 0.01	0.56 ± 0.02	-60.9 ± 0.7	-33.4 ± 1.7	54.3
30	D	1.58 ± 0.001	0.98 ± 0.49	-61.5 ± 0.4	-44.2 ± 15.2	53.4
31	S	0.76 ± 0.02	0.39 ± 0.02	-65.2 ± 0.1	-47.2 ± 0.8	52.8
33	S	29.55 ± 2.1	16.7 ± 7.4	-71.3 ± 0.5	-59.6 ± 4.6	54.7
34	S	0.2 ± 0.002	0.13 ± 0.01	-52.1 ± 0.4	-46.2 ± 0.8	55.5
41	S	0.36 ± 0.03	0.16 ± 0.03	-52.8 ± 0.2	-39.7 ± 3.4	55.3
42	S	0.28 ± 0.002	0.12 ± 0.001	-49.5 ± 0.1	-29.5 ± 0.1	54.6
42	D	0.22 ± 0.02	0.14 ± 0.001	-49.3 ± 0.1	-38.2 ± 1.0	54.5
43	S	0.26 ± 0.001	0.20 ± 0.03	-49.8 ± 0.02	-43.7 ± 2.1	53.8
43	D	0.31 ± 0.01	0.26 ± 0.001	-50.5 ± 0.03	-48.4 ± 0.7	53.5
44	S	4.99 ± 1.07	0.40 ± 0.32	-52.1 ± 5.5	15.7 ± 7.3	55.0
45	S	0.53 ± 0.02	0.08 ± 0.02	-58.4 ± 0.1	-14.2 ± 5.9	53.8
46	S	1.25 ± 0.04	0.20	-53.4 ± 0.01	-7.5	53.1

Table S2. Statistical results for Kendall's rank correlation between CH_4 response variables and canal chemistry and depth.

	**Dissolved oxygen**	**Canal depth**
**Response Variable**	Kendall's **τ**, p	Kendall's **τ**, p
**% oxidized**	0.34, 0.006	-0.20, 0.10
**Dissolved CH_4 (μM)**	-0.28, 0.02	0.26, 0.03
**$\delta^{13}C$-CH_4 (‰)**	0.33, 0.008	-0.19, 0.11

Table S3. Results for 1-way ANOVA testing the effect of vegetation.

Response	F	p	Vegetated	Open Water
**% oxidized**	$F(1,32) = 16.68$	< 0.001	83.1 ± 6.1	70.2 ± 11.9
**Dissolved CH_4 (µM)**	$F(1,32) = 5.9$	0.02	1.0 ± 0.9	4.5 ± 7.7
**$\delta^{13}C$-CH_4 (‰)**	$F(1,32) = 15.4$	< 0.001	-46.9 ± 7.7	-58.3 ± 9.3
**Dissolved oxygen (mg L^{-1})**	$F(1,19) = 2.4$	0.14	1.7 ± 0.28	1.5 ± 0.25
**Canal Depth (cm)**	$F(1,31) = 4.4$	0.04	35 ± 25	53 ± 26

Table S4. Results for 1-way ANOVA testing the effect testing the effect of land use types (smallholder mixed agriculture, smallholder plantation, industrial oil palm plantation, open undeveloped. The 1 canal in a secondary forest was omitted from statistical tests of land use due to insufficient number of observations for this land use).

Response	F	p
**% oxidized**	$F(3,29) = 0.34$	0.79
**Dissolved CH_4 (µM)**	$F(3,29) = 0.29$	0.83
**$\delta^{13}C$-CH_4 (‰)**	$F(3,29) = 0.58$	0.63
**Dissolved oxygen (mg L^{-1})**	$F(3,28) = 1.8$	0.17
**Canal Depth (cm)**	$F(3,28) = 1.8$	0.16

Table S5. Dissolved CH_4 and oxygen across peatland drainage canals across Southeast Asia.

Reference	Location	Season/Month	Land Uses[a]	Dissolved CH_4 (μM)[b]	Dissolved Oxygen (mg L^{-1})[b]
This Study	West Kalimantan	April/May	Mixed agriculture, oil palm, open undeveloped, smallholder plantation	3.0 ± 6.3 [0.05-31.6]	1.5 ± 0.5 [0.2-2.3]
Waldron et al. (2019)	Peninsular Malaysia	Dry	Degraded PSF	3.6 ± 6.5 [0.2-20.5]	2.0 ± 1.8 [0.1-4.9]
		Dry	Oil palm	0.9 ± 1.0 [0.04-2.4]	1.9 ± 1.2 [0.7-4.3]
Gandois et al. (2019)	West Kalimantan	January	Rubber, oil palm, mixed agriculture	–	2.3 ± 0.3 [1.9-2.7]
Kent (2019)	Central Kalimantan	Wet	Intact PSF	0.1-0.25	1.7
		Wet	Degraded PSF	0.7-3.0	3.5
		Dry	Intact PSF	0.7-3.4	2.2
		Dry	Degraded PSF	1.5-2.2	4.5
Thornton et al. (2018)	Central Kalimantan	Wet	Intact PSF	–	1.4 ± 0.2 [1.2-1.7]
Somers et al. (2023)	Brunei	January	Intact PSF	12.4 ± 11.8 [0.2 - 37.5]	–
		August	Intact PSF	12.9 ± 24.1 [0.02 - 85.7]	–

[a]PSF = peat swamp forest. [b]Values reported either as mean ± SD [range] when datasets were available; values from Kent (2019) reported as the range of median values observed across sampled canals from each land type and season (dissolved CH_4), or as the median per canal type (dissolved oxygen).

Table S6. CH_4 fluxes from peatland drainage canals across Southeast Asia. Data reported as mean ± standard deviation [range], except for Kent (2019) where values are reported as the median.

**Study**	**Location**	**Method**	**Flux (mg CH_4 m^{-2} d^{-1})**
This Study	West Kalimantan, Indonesia	Concentration-based; using k estimated from chambers	72.2 ± 151.2 [1.0-761.8]
		Manual chamber flux	94.9 ± 142.3 [1.0-542.9]
Jauhianunen & Silvennoinen (2012)	Central Kalimantan, Indonesia (settled)	Manual chamber flux	164 ± 328 [0-1311]
	Riau, Indonesia (settled)		1073 ± 1744 [0-5076]
	Riau, Indonesia (disturbed)		89 ± 169 [3-389]
Manning et al. (2019)	Sarawak, Malaysia	Manual chamber flux	135.9 ± 52.6 [-4130.7 - 5213.8]
Kent (2019)	Central Kalimantan, Indonesia (Intact, dry season)	Concentration-based; using k estimated from chambers	10.4
	Central Kalimantan, Indonesia (Intact, wet season)		2.8
	Central Kalimantan, Indonesia (Degraded, dry season)		39.6
	Central Kalimantan, Indonesia (Degraded, wet season)		17.8
Swails et al. (2024)	Jambi, Indonesia (oil palm)	Manual chamber flux	4.5 ± 1.97
Grinham (unpublished)*	Solomon Islands	Manual chamber flux	383.1

*Data reported in Peacock et al. (2021).

Supplementary References:

Cole, J. J. & Caraco, N. F. Atmospheric exchange of carbon dioxide in a low-wind oligotrophic lake measured by the addition of SF_6. *Limnology & Oceanography* **43**, 647–656 (1998).

Gandois, L. *et al.* From canals to the coast: dissolved organic matter and trace metal composition in rivers draining degraded tropical peatlands in Indonesia. *Biogeosciences* **17**, 1897–1909 (2020).

Holgerson, M. A., Farr, E. R. & Raymond, P. A. Gas transfer velocities in small forested ponds. *J. Geophys. Res. Biogeosci.* **122**, 1011–1021 (2017).

Jauhiainen, J. & Silvennoinen, H. Diffusion GHG fluxes at tropical peatland drainage canal water surfaces. *Suoseura* **63**, 93–105 (2012).

Kent, M. S. Greenhouse gas emissions from channels draining intact and degraded tropical peat swamp forest. (The Open University, 2019).

Manning, F. C., Kho, L. K., Hill, T. C., Cornulier, T. & Teh, Y. A. Carbon Emissions From Oil Palm Plantations on Peat Soil. *Front. For. Glob. Change* **2**, 37 (2019).

Peacock, M. *et al.* Global importance of methane emissions from drainage ditches and canals. *Environ. Res. Lett.* **16**, 044010 (2021).

Somers, L. D. *et al.* Processes Controlling Methane Emissions From a Tropical Peatland Drainage Canal. *JGR Biogeosciences* **128**, e2022JG007194 (2023).

Swails, E. *et al.* Soil nitrous oxide and methane fluxes from a land-use change transition of primary forest to oil palm in an Indonesian peatland. *Biogeochemistry* **167**, 363–381 (2023).

Taillardat, P. *et al.* Carbon Dioxide and Methane Dynamics in a Peatland Headwater Stream: Origins, Processes and Implications. *JGR Biogeosciences* **127**, e2022JG006855 (2022).

Thornton, S. A., Dudin, Page, S. E., Upton, C. & Harrison, M. E. Peatland fish of Sebangau, Borneo: diversity, monitoring and conservation. *Mires and Peat* 1–25 (2018) doi:10.19189/MaP.2017.OMB.313.

Waldron, S. *et al.* C mobilisation in disturbed tropical peat swamps: old DOC can fuel the fluvial efflux of old carbon dioxide, but site recovery can occur. *Sci Rep* **9**, 11429 (2019).
