# Supplementary material for: Fate of methane in canals draining tropical peatlands
Source: Nat Commun. 2024 Nov 11;15:9766. doi: 10.1038/s41467-024-54063-x (PMC11555057; doi:10.1038/s41467-024-54063-x)
Supplement: Supplementary file 2 — Description of Additional Supplementary Files [file 41467_2024_54063_MOESM2_ESM.pdf]

Description of Additional Supplementary Files

File Name: Supplementary Data 1
Description: Overview of data collected at 34 canal sampling sites in West Kalimantan, Indonesia.

File Name: Supplementary Data 2
Description: Peat porewater CH4 concentration and δ13C-CH4 measurements from 6 profiles collected adjacent to a subset of studied drainage canals.
